# Incidence of a Multicomponent Physical Exercise Program on Body Composition in Overweight or Obese People Aged 60 Years or Older from Chile

**DOI:** 10.3390/jfmk9020081

**Published:** 2024-04-24

**Authors:** Yazmina Pleticosic-Ramírez, Marcos Mecías-Calvo, Víctor Arufe-Giráldez, Rubén Navarro-Patón

**Affiliations:** 1 Departamento de Salud, Universidad Internacional Iberoamericana, Campeche 24560, Mexico; yazmina.pleticosic@doctorado.unini.edu.mx; 2 Facultad de Educación, Pedagogía en Educación Física, Universidad San Sebastián, Lientur 1457, Concepción 4080871, Chile; 3 Facultade de Formación do Profesorado, Universidade de Santiago de Compostela, 27001 Lugo, Spain; marcos.mecias@usc.es (M.M.-C.); ruben.navarro.paton@usc.es (R.N.-P.); 4 Facultad de Ciencias de la Educación, Universidad de A Coruña, 15008 A Coruña, Spain

**Keywords:** body mass index, percentage of fat mass, percentage of muscle mass, percentage of visceral fat, physical activity programs

## Abstract

This research aimed to explore the changes produced in body mass index (BMI), fat mass percentage (FMP), muscle mass percentage (MMP), and visceral fat percentage (VFP) in 60-year-old or over overweight or obese people after a multicomponent exercise program. This quasi-experimental study involved 70 overweight or obese older people between 60 and 86 years old (M = 73.15; SD = 5.94) who were randomly assigned to a control group (CG, n = 35) and an experimental group (EG, n = 35). At the beginning and at the end of the intervention program, anthropometric and body composition data were collected. The results showed an increase in BMI after the intervention in the CG (*p* = 0.010) and a decrease in the EG (*p* < 0.001). The results regarding the FMP indicate a significant decrease in the EG (*p* < 0.001) after the intervention, as occurs with the VFP (*p* = 0.003). The MMP increased in the EG (*p* < 0.001) after the intervention program. Regarding gender, statistically significant differences were found in the MMP after the intervention (*p* = 0.025), with higher percentages in men in the EG. VFP decreased in both men (*p* = 0.005) and women (*p* = 0.019) in the EG. From the results obtained, we can say that a 6-month multicomponent program produces a decrease in BMI, FMP, and VFP and an increase in MMP in its participants. This type of intervention seems to produce a greater increase in muscle mass in men than in women and a decrease in VFP in both genders.

## 1. Introduction

The global phenomenon of population ageing presents significant challenges for public health, particularly with regard to the management of overweight and obesity in older people [1]. By 2030, the elderly population will represent 20% of the world’s population, and half of them will suffer from obesity [2]. This demographic shift not only affects the distribution of age groups but also poses unique health challenges, especially in addressing the prevalence of overweight and obesity among older adults [3,4].

Overweight and obesity are multifactorial chronic health conditions [5,6] that affect a substantial proportion of the world’s population, characterised by excess body fat [5,7], and their prevalence increases with age [8]. In older people, excess weight can have important health consequences, including increased joint loading, increased risk of chronic diseases, and decreased quality of life [9]. Furthermore, obesity in old age is associated with greater vulnerability to functional disability and a higher mortality rate [9].

Addressing the health implications of overweight and obesity in older adults has led to the emergence of multicomponent physical exercise programs as effective strategies [10,11]. Villa-González et al. [12] demonstrated that “the combination of aerobic exercise and strength can have positive effects on weight loss, body composition, and cardiovascular health in adults with severe obesity”. Similarly, López et al. [13] found that programs incorporating resistance training are effective in improving body composition and weight in overweight or obese individuals. These findings underscore the importance of tailored exercise interventions to improve physical health and address weight-related challenges in older populations [14].

The importance of physical exercise to promote the health and well-being of older people has been widely recognised [11,15]. According to recent studies [16], regular exercise not only improves muscle strength, cardiovascular function, and bone health but also plays a crucial role in the prevention and control of chronic diseases, improves sleep quality, and reduces the risk of depression and anxiety. This aligns with the statement that “ageing has become a major global public health issue due to the significant increase in the number of older people” [17].

In conclusion, the intersection of population ageing and the prevalence of overweight and obesity presents a critical public health problem worldwide. As highlighted, “the prevalence of overweight and obesity in old age has increased dramatically around the world, posing serious challenges to public health and healthcare systems” [8]. Understanding the impact of these factors on health outcomes and implementing targeted interventions, such as multicomponent exercise programs, are essential to address the complex health needs of older adults facing weight-related challenges [12]. These benefits extend to improvements in body composition, percentage of fat and muscle mass, as well as percentage of visceral fat mass, suggesting that exercise may be an effective tool in the management of these conditions.

For all of the above, the aim of this study was to determine the effect of a multicomponent physical exercise program on body composition in adults over 60 years of age who are overweight or obese. Specifically, we seek to quantify the effects of physical exercise on variables such as BMI, fat mass, lean mass, and the percentage of visceral fat mass in this population.

## 2. Materials and Methods

### 2.1. Design and Participants

For this quasi-experimental study with a control group [18], a total of 153 people, both men (59) and women (94), aged 60 years or older, who were overweight or obese were included. The subjects belonged to clubs of the Regional Federation of Community Unions of older people in the Biobío region of the city of Concepción (Chile) and were selected in a non-probabilistic manner and by convenience, according to the subjects to whom access was had. The inclusion criteria were: (a) being 60 years or older; (b) being overweight or obese; (c) not suffering from any illness that prevents participation in the tests or intervention program; (d) physical independence; (e) being able to sign the informed consent; and (f) being able to participate in the entire process (i.e., initial data collection, exercise program (80%), and final data collection).

**Figure 1:**
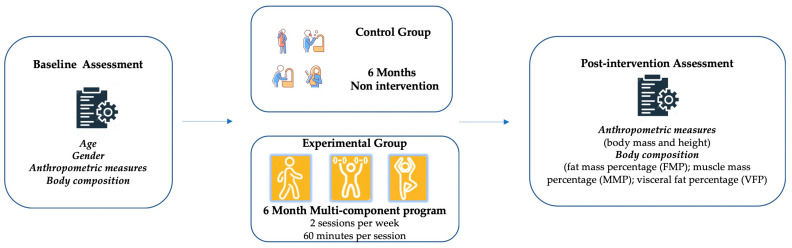
Study design.

Finally, 70 subjects met all the inclusion criteria and were randomised into a control group (CG, n = 35; 33 women/2 men) and an experimental group (EG, n = 35; 28 women/7 men) (Figure 1).

### 2.2. Study Variables (Table 1)

Dependent variables:

(a) Anthropometric measurements: “study of size, proportion, maturation, body shape and composition, and functions of the organism, with the objective of describing physical characteristics, evaluating and monitoring growth, nutrition, and the effects of physical activity” [19]. For this study, anthropometric measurements, such as weight and height, were evaluated to determine the state of overweight or obesity through BMI.
(b) Body composition: “allows to quantify body reserves of the organism and thus detect and correct nutritional problems such as obesity situations” [20]. In older people, the increase in fat mass and the reduction of lean mass is highlighted [21]. For this study, the percentage of fat mass, percentage of muscle mass, and percentage of visceral fat will be identified.

Independent variables:

(a) Sex: Man/Woman
(b) Group: CG/EG

### 2.3. Instruments

#### 2.3.1. Questions Regarding Sex (Male/Female) and Age

Using these questions, the variables age (years) and sex (male/female) were collected.

#### 2.3.2. Anthropometric and Body Composition Measurements

For anthropometric and body composition measurements, the protocol of the International Society for the Advancement of Kinanthropometry (ISAK) [22] was used for both body mass and height. These two measurements allowed us to determine the degree of obesity through the body mass index (BMI) with the formula [weight kg/height m^2^], following the WHO measurements [23].

Height measurement was performed with the portable SECA 206 stadiometer in the maximum extension position, placing the square firmly on the vertex, compressing the hair as much as possible, and asking the person to inhale deeply and hold their breath before they exhaled [22].

Body mass was calculated using the Omron HBF−514C (Omron Healthcare, Inc., Chicago, IL, USA) equipment. Weight was evaluated with minimal clothing, checking that the scale was at zero. These measurements were routinely performed in the morning, twelve hours after the last meal [22].

To estimate body composition and obtain fat mass (FMP), percentage of muscle mass (MMP) and percentage of visceral fat (VFP), electrical bioimpedance was used with the Omron HBF−514C (Omron Healthcare, Inc., Chicago, IL, USA) equipment, with a maximum capacity of 150 kg and precision of 100 grammes. This measurement was performed with the participant barefoot, with light clothing, and without metal accessories.

#### 2.3.3. Intervention Program

No physical exercise program was applied to the CG, with participants allowed to continue with their usual activity. A multicomponent physical exercise program was applied to the EG that lasted 6 months (2 sessions/week), with each session lasting 60 min each. Each session was organised as follows: warm-up (10 min); main part (40 min); cool-down (10 min, Table 2). Participants worked on different contents within each phase of the session (warm-up—general mobility, balance, and agility; main part—aerobic endurance and resistance training; cool-down—stretching). All sessions of the multicomponent program were taught by the principal researcher, a graduate in physical education with 15 years of experience.

### 2.4. Procedures

Firstly, contact was made with the management of the clubs of the Regional Federation of Community Unions of older people in the Biobío region of the city of Concepción (Chile) to explain what the objective of the research was. After obtaining management approval, potential participants were contacted through an invitation letter and information meetings explaining the objective, purpose, design, study procedures, confidentiality statement, and voluntary participation.

After obtaining signed informed consent, the necessary sociodemographic data (age and gender) were recorded, and anthropometric and body composition measurements were obtained prior to the start of the physical exercise program for the experimental and control groups. To perform body composition measurements, participants were instructed to meet the following prerequisites: no oedema; fasting from food and drinks for 4 h before the exam time; abstaining from consuming alcoholic beverages the day before the exam; avoiding excessive consumption of foods rich in caffeine (chocolates, dark teas, and coffee) in the two days before the exam; abstaining from performing intense physical activity or using a sauna the day before the exam; urinating 30 min before the procedure.

In one day, between 9 a.m. and 12 p.m., using standardised and calibrated equipment applied by trained evaluators, data were collected using questionnaires regarding information such as sociodemographic background and anthropometric and body composition evaluations. After collecting the initial data, the intervention program was applied for 6 months with a weekly frequency of 2 sessions and 60 min per session. Data collection (anthropometric and body composition measurements) was carried out one week after completion of the intervention programs for both the CG and the EG. This data collection was carried out again, replicating the conditions of the initial data collection.

All research was carried out in accordance with the Declaration of Helsinki. The research protocol was sent to and approved by the Ethics Committee of the Universidad Internacional Iberoamericana on 22 June 2022 with code number CR-163.

### 2.5. Statistical Analysis

The results of the quantitative variables (i.e., anthropometry, body composition, and age) are presented through measures of central tendency (mean and standard deviation), and for the qualitative variables (i.e., gender, and degree of overweight or obesity), percentages and frequencies. To verify the normality of the data, the Kolmogorov–Smirnov test was used. First, the descriptive statistics (mean and standard deviation of the mean) were calculated for each dependent variable examined. Secondly, the Chi square test was performed to check whether the groups (CG vs. EG) were equivalent with respect to gender and the degree of obesity of the participants, and an independent samples t-test was performed to check the equivalence between the groups in terms of age, anthropometry variables, and body composition. After 6 months of the intervention, a three-factor ANOVA was performed [i.e., time (pre-test vs. post-test), group (control group vs. experimental group), and gender (male vs. female)], using time as a repeated measures factor. To analyse the possible effect of these factors on the variables of anthropometry and body composition, and their interaction, the Bonferroni statistic was used and the effect size was calculated in terms of eta squared (η^2^), considering the effect size small (0.01), medium (0.06) or large (0.14 or higher). Statistical treatment of the data was carried out using the IBM SPSS Statistics program for Windows, version 25.0 with a significance level of *p* < 0.05.

## 3. Results

A total of 70 overweight or obese older people [9 men (12.9%) and 61 women (87.1%)] aged between 60 and 85 (M = 71.14; SD = 5.95) who met the inclusion criteria participated in this study. They were randomised into a control group consisting of 35 participants (33 women and 2 men) and an experimental group, also consisting of 35 participants (28 women and 7 men).

### 3.1. Basic Characteristics of the Sample

Once the tests were applied to verify that the groups (CG and EG) were homogeneous, the results obtained show that there were no statistically significant differences in any of the variables [i.e., mean age (*p* = 0.391); gender (*p* = 0.075); average height (*p* = 0.685); average weight (*p* = 0.443); BMI (*p* = 0.215); fat mass (*p* = 0.612); muscle mass (*p* = 0.482); and degree of overweight/obesity (*p* = 0.528)]. For this reason, it can be said that both groups were equivalent (Table 3).

### 3.2. Anthropometry and Body Composition of the CG vs. EG

The following are the findings from the comparisons of the anthropometry and body composition between the CG and the EG before and after the multicomponent exercise program was implemented (Table 4):

#### 3.2.1. Body Mass Index (BMI)

Regarding BMI, the results indicate that there were statistically significant interactions in the comparison of the factors group (CG vs. EG) × time (before vs. after). In the CG [F(1, 66) = 6.939, *p* = 0.010, η^2^ = 0.095, 95% CI −1.039, −0.143], before (M = 30.71, SD = 4.07) and after the intervention (M = 30.94, SD = 3.99), the results indicate that this group had a higher BMI after 6 months. Statistically significant interactions were also found in the EG [F(1, 66) = 16.936, *p* < 0.001, η^2^ = 0.204, 95% CI 0.276, 1.039] with a large effect size, but in this case the BMI was lower after the intervention [before (M = 31.88, SD = 3.73); after (M = 31.20, SD = 3.74)]. No statistically significant interactions were found between the CG and EG, neither before (*p* = 0.408) nor after (*p* = 0.26) the application of the multicomponent physical exercise program.

Finally, a significant interaction of the factors group × gender × time was found between women (M = 30.60, SD = 3.76) and men (M = 36.50, SD = 4.94) of the CG [F(1, 66) = 4.537, *p* = 0.037, η^2^ = 0.064, (95% CI −11.419, −0.369)] after the 6-month period, with a lower BMI in women than in men. No statistically significant interactions were found in the rest of the comparisons.

#### 3.2.2. Fat Mass Percentage (FMP)

The results obtained in the FMP variable indicate that there was a statistically significant interaction of the group factor and time in the EG [F(1, 66) = 42.083, *p* < 0.001, η^2^ = 0.389, 95% CI 1.075, 2.032], which presents a higher FMP before (M = 45.54, SD = 7.85) than after (M = 43.91, SD = 7.66) the intervention, with a large effect size. There were no differences in the CG (*p* = 0.244). No statistically significant interactions were found comparing the CG with the EG before (*p* = 0.776) or after (*p* = 0.171) the intervention.

The results of the contrast of the interaction between the factors gender (man vs. woman) × time (pre-intervention vs. post-intervention) × group (CG vs. EG) indicated statistically significant differences between women in the CG and EG before the intervention [F(1, 66) = 8.277, *p* = 0.004, η^2^ = 0.081, 95% CI −5.905, −1.160], with a higher FMP in women of the EG (M = 48.71, SD = 4.59) than those of the CG (M = 45.18, SD = 4.66). These significant differences disappeared after the intervention (*p* = 0.104). Significant differences were also found between men from the CG and EG [F(1, 66) = 3.972, *p* = 0.050, η^2^ = 0.056, 95% CI 0.053, 14.196] after the intervention, with a higher FMP in men of the CG (M = 38.50, SD = 4.94) than those of the EG (M = 31.42, SD = 4.57).

#### 3.2.3. Muscle Mass Percentage (MMP)

Regarding MMP, the results indicate that there was a statistically significant interaction of the group factor and time in the EG [F(1, 66) = 20.012, *p* < 0.001, η^2^ = 0.233, 95% CI −1.240, −0.475] with a large effect size, with a lower MMP before (M = 22.45, SD = 3.45) than after (M = 23.22, SD = 3.54) the intervention. There were no differences in the CG (*p* = 0.265). No statistically significant interactions were found when comparing the CG with the EG before (*p* = 0.802) or after (*p* = 0.089) the intervention.

The results of the contrast of the interaction between the factors gender (man vs. woman) × time (pre-intervention vs. post-intervention) × group (CG vs. EG) indicated statistically significant differences between women in the CG and EG before the intervention [F(1, 66) = 11.405, *p* = 0.001, η^2^ = 0.147, 95% CI 0.704, 2.740], with a higher MMP in women of the CG (M = 22.75, SD = 2.06) than those of the EG (M = 21.03, SD = 1.87) with a large effect size. These significant differences disappeared after the intervention (*p* = 0.137). These differences were also found between men from the CG and EG [F(1, 66) = 5.263, *p* = 0.025, η^2^ = 0.074, 95% CI −6.813, −0.473] after the intervention, with a higher MMP in men of the EG (M = 29.14, SD = 2.54) than those of the CG (M = 25.50, SD = 2.12). These differences did not exist before the intervention (*p* = 0.183).

A significant interaction of the factors group × gender × time was also found between the women and men of the CG, both before the intervention [F(1, 66) = 5.035, *p* = 0.028, η^2^ = 0.073, 95% CI −6.128, −0.357] and after [F(1, 66) = 4.284, *p* = 0.042, η^2^ = 0.060, 95% CI −5.864, −0.105]. In the EG, statistically significant interactions were also found both before [F(1, 66) = 71.837, *p* < 0.001, η^2^ = 0.499, (95% CI −8.781, −5.433] and after the intervention [F(1, 66) = 78.039, *p* < 0.001, η^2^ = 0.511, 95% CI −9.064, −5.722] with large effect sizes. In all the previous cases, the MMP was higher in men.

Finally, a significant interaction of the factors group × gender × time was found in the women of the EG when they were compared before (M = 21.03, SD = 1.87) and after (M = 21.75, SD = 1.73) the intervention [F(1, 66) = 17.372, *p* < 0.001, η^2^ = 0.208, 95% CI −1.056, −0.372], showing an increase of the MMP after the intervention program with a large effect size. These same results were obtained in the men of the EG before (M = 28.14, SD = 2.11) and after (M = 29.14, SD = 2.54) the intervention [F(1, 66) = 8.512, *p* = 0.005, η^2^ = 0.114, 95% CI −1.684, −0.316] with a medium effect size. No statistically significant interaction was found in the comparison between women (*p* = 0.129) or men (*p* = 0.438) of the CG.

#### 3.2.4. Visceral Fat Percentage (VFP)

Regarding the VFP, the results indicate that there was a statistically significant interaction of the group factor and time between the CG and EG participants before the intervention [F(1, 66) = 4.513, *p* = 0.037, η^2^ = 0.064, CI 95% 0.138, 4.435], with a greater VFP in the CG (M = 13.14, SD = 3.97) than in the EG (M = 14.91, SD = 3.45). These differences decreased after the intervention [F(1, 66) = 7.497, *p* = 0.008, η^2^ = 0.102, 95% CI 0.822, 5.250], although the VFP was significantly reduced in the EG. Significant differences were found when comparing the EG before vs. after the intervention [F(1, 66) = 9.247, *p* = 0.003, η^2^ = 0.090, 95% CI −1.399, −0.601], with a higher VFP before (M = 14.95, SD = 3.45) than after (M = 14.25, SD = 3.31). No statistically significant interaction was found before and after the intervention in the CG (*p* = 0.471).

The results of the contrast of the interaction between the factors gender (man vs. woman) × time (pre-intervention vs. post-intervention) × group (CG vs. EG) indicate the existence of statistically significant differences between women in the CG and EG before the intervention [F(1, 66) = 4.262 *p* = 0.043, η^2^ = 0.061, 95% CI −2.668, −0.045], with lower VFP in the women of the CG (M = 12.39, SD = 2.43) than those of the EG (M = 13.75, SD = 2.41). These significant differences disappeared after the intervention (*p* = 0.175). These differences were also found between men from the CG and EG before [F(1, 66) = 8.366, *p* = 0.005, η^2^ = 0.112, 95% CI −6.813, −0.473], with a medium effect size, and after the intervention [F(1, 66) = 10.984, *p* = 0.001, η^2^ = 0.143, 95% CI 2.783, 11.217], with a large effect size, with a lower VFP in men of the EG, both before and after the intervention.

Significant interactions of the factors group × gender × time were also found between the women and men of the CG, both before the intervention [F(1, 66) = 49.561, *p* < 0.001, η^2^ = 0.429, 95% CI—16.823, −9.389] and after [F(1, 66) = 43.184, *p* < 0.001, η^2^ = 0.396, 95% CI −16.436, −8.776] with a large effect size. In the EG, statistically significant interactions were also found both before [F(1, 66) = 29.038, *p* < 0.001, η^2^ = 0.306, (95% CI −7.978, −3.665] and after the intervention [F(1, 66) = 17.665, *p* < 0.001, η^2^ = 0.211, 95% CI −6.901, −2.456], also with a large effect size. In all the previous cases, the VFP was higher in men.

Finally, a significant interaction of the factors group × gender × time was found in the women of the EG when compared before (M = 13.75, SD = 2.41) and after (M = 13.32, SD = 2.40) the intervention [F(1, 66) = 5.476, *p* = 0.019, η^2^ = 0.080, 95% CI 0.072, 0.786], with a medium effect size, showing a decrease of the VFP after the intervention program. The same results were obtained in the men of the EG before (M = 19.57, SD = 3.15) and after (M = 18.00, SD = 3.95) the intervention [F(1, 66) = 19.313, *p* = 0.005, η^2^ = 0.226, 95% CI 0.858, 2.285] with a large effect size. No statistically significant interaction was found in the comparison between the CG women (*p* = 1.00) or between men (*p* = 0.457).

## 4. Discussion

The motivation to carry out this study arose from the observation of the effects that different types of physical exercise have on body composition in older people. Therefore, an attempt was made to implement a multicomponent exercise program whose objective was to determine the effects of multicomponent exercise on the BMI, fat mass percentage, muscle mass percentage, and visceral fat percentage of overweight or obese people 60 years of age or over. Overall, the results of this study show that there was a significant decrease in the BMI, fat mass percentage (FMP), and visceral fat percentage (VFP) in the group of older people who followed the multicomponent physical exercise program for 6 months. Furthermore, the results also indicate that there was a significant increase in the muscle mass percentage (MMP) in this group.

In more detail, in relation to the results obtained regarding the BMI, as revealed by some systematic reviews [11,24], people with overweight or obesity who participated in exercise interventions achieved a reduction in BMI compared to people who did not participate in exercise interventions. The results obtained in this study show that, after 6 months of the multicomponent exercise intervention, the BMI suffered variations, both in the CG (increasing significantly) and in the EG (decreasing significantly) [24]. In relation to the results obtained in our study, previous research was found, such as that developed by Vargas and Rosas [25], where an exercise program was also applied for six months of intervention with a frequency of three times per week, as in our study, and produced a decrease in the BMI of the people who participated in the experimental group. These results obtained are similar to those reported in previous research [26,27,28], in which significant improvements in BMI were shown after specific interventions such as the one carried out in the study, although in some cases the intervention period was shorter [28], or with other types of physical exercise programs such as moderate aerobic training [26] or vigorous interval training [27]. After the 6-month period, men in the CG had an increase in their BMI, while women in the EG had a decrease in their BMI. These findings are in line with previous studies that showed variations in BMI according to sex and the type of physical exercise intervention performed [29]. Furthermore, the results show a lower BMI in women than in men, thus supporting the hypothesis that gender may have something to do with the response to physical exercise and changes in BMI [29] that favour women. Finally, regarding BMI, the results show that the multicomponent physical exercise intervention program had a significant and positive effect on the BMI of the participants, with notable differences between groups (CG vs. EG), sex (man vs. woman), and time periods (before vs. after the intervention). These results coincide with previous research on the connection between anthropometric factors, BMI variations, and physical exercise, since participating in a physical exercise program improves BMI (reducing it) compared to those who did not participate [28,29].

The results regarding FMP revealed that, unlike the CG, the EG had a significantly lower FMP after the intervention, as has occurred in similar studies where a resistance exercise program was applied for 12 weeks [30], with elastic bands [31,32] for 8 weeks [33]. Specifically, the results of the FMP prior to the intervention were higher in the women of the EG than those of the CG. After the intervention, these differences disappeared. These variations were also observed after the intervention between the CG and EG men, where the CG men had a higher FMP. This could be because in an older adult population that does not practice physical activity or other training programs, the FMP does not reduce and may even increase [34,35]. Furthermore, both the CG and EG women had a higher FMP than men before and after the intervention. The importance of these findings lies in the fact that for physical exercise programs for overweight or obese older people to have successful development in terms of weight control and health, they must be adapted to each individual, taking into account gender and type of physical exercise for the modification of body composition [13].

In reference to the MMP results, a significant increase in this variable was observed after the intervention in the EG. In this sense, scientific research developed in this area obtains similar results, stating that with the implementation of an exercise program based on HIT [35] or aerobic training [36], there is an increase in muscle mass as has occurred in the group that participated in the multicomponent program.

On the contrary, no variations were observed in the CG. More specifically, the CG women had a higher percentage of muscle mass than the EG women before the multicomponent physical exercise program. However, these differences in MMP disappeared after the intervention. Regarding men, differences were observed between those from the CG and the EG after the application of the physical exercise program, with the men from the EG having a higher MMP than the men from the CG [30,32,33], a difference that did not exist before the intervention. Furthermore, both before and after the intervention, in both the CG and the EG, men had a higher MMP than women. This may be because interventions in obese older people result in fewer favourable changes in the body than those in people with normal weight [30]. These findings, again, are important from the point of view of the design of specific and personalised exercise programs for the population of this study, and variables such as gender, duration, and type of intervention are taken into account in order to achieve better efficiency, since both longer and shorter programs have a positive effect on this variable [37]. However, in terms of muscle health and body composition, the increase in the percentage of lean and muscle mass does not always translate into higher quality or better physical performance in overweight and obese older people [38].

Finally, the results of the study regarding VFP indicate that the EG participants significantly decreased their visceral fat after the intervention [36,39,40]. These results indicate that the multicomponent exercise program reduced the VFP of the EG participants, as occurs with other types of training such as vigorous interval training [27] or aerobic [36,40], intense aerobic [39], HIT [39] or strength training [40]. Specifically, women in the CG had a lower VFP than women in the EG before the intervention. These differences disappeared after the intervention since an exercise program helps with the loss of VFP even without a diet [41,42]. In both groups (CG and EG), the VFP of men was higher than that of women as in the study of Yuan, Chang, and Wang [43], where there was also an association between visceral fat and “frailty” in older adults.

As an important limitation of the study, we would like to highlight the low participation of men in the program, which means that the results obtained in this group must be taken with caution.

## 5. Conclusions

The results of the study demonstrate the capacity of a multicomponent physical exercise program to reduce the values of BMI, FMP, and VFP in the experimental group. These findings highlight the importance of developing individualised programs that take into account the particularities of each person to obtain the best possible results in body composition and muscle health.

Furthermore, the findings of the study show that MMP increased significantly in both men and women after the intervention in the group that carried out the multicomponent exercise program, indicating that such a program is effective for increasing muscle mass in people over 60 years of age who are overweight or obese.

However, all the results obtained in men should be taken with caution due to the small number of men who participated in the study.

## Figures and Tables

**Table 1:** Study variables.

Variable	Definition	Instrument	Measurement Units
**Age**	How long the person has lived	Sociodemographic questionnaire	Years
**Sex**	Male or female organic condition	Sociodemographic questionnaire	Man/Woman
**Body Mass Index**	Relationship between weight and height	Height and weight	Kilograms/m^2^
**Fat mass**	Total fat present in the body	Omrom HBF 514 Electric Bioimpedance Scale	% body fat
**Muscle Mass**	Total muscle tissue in the body	Omrom HBF 514 Electric Bioimpedance Scale	% muscle mass
**Visceral Fat**	Total fatty tissue in internal organs	Omrom HBF 514 Electric Bioimpedance Scale	% visceral fat
**Physical Exercise Program**	Multicomponent physical exercise that includes strength, resistance, flexibility and balance	Sessions	% attendance

**Table 2:** Phases and contents of the sessions and types of exercises of the multicomponent program.

Phases	Content	Typical Exercises
(1) Warm-up (10 min)	(1.1.) General mobility (1.2) Balance (1.3) Agility	1.1. Walking forward/reverse/lateral. 1.1. Walking with different movements of the upper extremities. 1.2. Static balancing on one leg. 1.2. Unstable sitting balance. 1.3. Walking quickly for a distance of 5 m. 1.3. Walking in a zigzag while bouncing a ball.
(2) Main part (40 min)	(2.1.) Aerobic endurance (2.2) Resistance training: (2 sets of 10 to 15 repetitions/8–10 exercises)	2.1. Entertaining dance/walk/low-impact aerobics. 2.2. Upper and lower body exercises for large muscle groups with elastic resistance bands; biceps, triceps, deltoids, wrist flexors and extensors, pectorals, quadriceps, hamstrings, gastrocnemius, glutes, adductors, abductors, etc. 2.3. Core exercises on a mat; abdominals, dorsals, obliques, transverse abdominis.
(3) Cool-down (10 min)	(3.1) Stretching: 2 series with maintenance of 30 s in each task	3.1. 8–10 tasks: stretching biceps, triceps, deltoids, wrist flexors and extensors, pectorals, quadriceps, hamstrings, gastrocnemius, glutes, adductors, and iliopsoas.

**Table 3:** Sample characterization.

	Control Group	Experimental Group
**Variables**		
**Average age (years)**	72.54 ± 5.55	73.77 ± 6.32
**Sex** Man Woman	2 (72.2%) 33 (27.8%)	7 (27.8%) 28 (72.2%)
**Average height (m)**	1.538 ± 7.16	1.530 ± 9.16
**Average weight (Kg)**	72.51 ± 11.99	74.80 ± 12.75
**Average BMI (Kg/m^2^)**	30.71 ± 4.07	31.88 ± 3.73
**Fat mass (%)**	44.74 ± 4.94	45.54 ± 7.85
**Muscle mass (%)**	22.94 ± 2.15	22.45 ± 3.45
**Degree of overweight/obesity** Overweight Type I Obesity Type II Obesity Type III Obesity	17 (24.3%) 13 (18.6%) 4 (5.7%) 1 (1.4%)	11 (15.7%) 17 (24.3%) 6 (8.6%) 1 (1.4%)

Note: Quantitative variables are expressed as mean and standard deviation, and qualitative variables are expressed as frequencies and percentages.

**Table 4:** Anthropometry and body composition outcomes according to group (CG/EG) and gender (Men/Women).

Variable		CG Pre (n = 35)	EG Pre (n = 35)	CG Post (n = 35)	EG Post (n = 35)
**Body Mass Index** **(km/m^2^)**	women	30.42 ± 3.92	32.07 ± 3.99	30.60 ± 3.76 †	31.28 ± 4.01
	men	35.50 ± 4.94	31.14 ± 2.54	36.50 ± 4.94	30.85 ± 2.54
	all	30.71 ± 4.07 ***	31.88 ± 3.73 *	30.94 ± 3.99	31.20 ± 3.74
**Fat mass** **(%)**	women	45.18 ± 4.66 **	48.71 ± 4.69	45.15 ± 4.50	47.03 ± 4.34
	men	37.50 ± 4.94	32.85 ± 3.97	38.50 ± 4.94 *	31.42 ± 4.57
	all	44.74 ± 4.94	45.54 ± 7.85 *	44.77 ± 4.71	43.91 ± 7.66
**Muscle mass** **(%)**	women	22.75 ± 2.06 †,**	21.03 ± 1.87 †,*	22.51 ± 2.04 †,*	21.75 ± 1.73 †
	men	26.00 ± 1.41	28.14 ± 2.11 *	25.50 ± 2.12 *	29.14 ± 2.54
	all	22.94 ± 2.15	22.45 ± 3.45 *	22.68 ± 2.13	23.22 ± 3.54
**Visceral fat** **(%)**	women	12.39 ± 2.43 †,**	13.75 ± 2.41 †,*	12.39 ± 2.34 †	13.32 ± 2.40 †
	men	25.50 ± 4.94 **	19.57 ± 3.15 *	25.50 ± 5.65 *	18.00 ± 3.95
	all	13.14 ± 3.97 **	14.91 ± 3.45 *	13.11 ± 3.86	14.25 ± 3.31

Note: * *p* < 0.05 different to post-intervention EG; ** *p* < 0.05 different to pre-intervention EG; *** *p* < 0.05 different to post-intervention CG; † *p* < 0.05 different to men.

## Data Availability

The datasets presented in this article are not available because they are part of a doctoral thesis which has not yet been defended. Requests to access the datasets should be directed to Y.P.-R.
